# Diabetes distress among caregivers of adolescents with type 1 diabetes

**DOI:** 10.1093/jpepsy/jsaf051

**Published:** 2025-07-11

**Authors:** Tabitha McCarty, Hailey Inverso, Randi Streisand, Sydney Garretson, Emma Straton, Nkemjika Okonkwo, Sarah S Jaser

**Affiliations:** Department of Pediatrics, Vanderbilt University Medical Center, Nashville, TN, United States; Psychology and Behavioral Health, Children’s National Hospital, Washington, DC, United States; Psychology and Behavioral Health, Children’s National Hospital, Washington, DC, United States; Department of Psychiatry and Behavioral Health, George Washington School of Medicine & Health Sciences, Washington, DC, United States; Department of Pediatrics, Vanderbilt University Medical Center, Nashville, TN, United States; Psychology and Behavioral Health, Children’s National Hospital, Washington, DC, United States; Department of Pediatrics, Vanderbilt University Medical Center, Nashville, TN, United States; Department of Pediatrics, Vanderbilt University Medical Center, Nashville, TN, United States

**Keywords:** diabetes, caregiver functioning, parent psychosocial functioning, adolescents

## Abstract

**Objective:**

Diabetes distress (DD), the negative emotional response related to the burdens of diabetes management, has been studied primarily in adults with type 2 diabetes and youth with type 1 diabetes (T1D), but less is known about DD among caregivers of youth with T1D.

**Methods:**

Caregivers of adolescents with T1D (*n *= 198, *M*_age_ = 45.6 ± 7.3, 84% female, 66% non-Hispanic White) were enrolled in a two-site randomized clinical trial aimed at treating DD among adolescents. The current study is a secondary analysis of baseline data to examine factors associated with DD among caregivers. Caregivers completed measures of DD, diabetes-related family conflict, and adolescents’ diabetes self-management behaviors. Caregivers also reported on demographic factors, and clinical data were extracted from adolescents’ medical records.

**Results:**

Female caregivers, caregivers of younger adolescents, caregivers reporting lower household income, caregivers of lower subjective social status, and single/nonpartnered caregivers reported significantly higher caregiver DD. Further, after adjusting for demographic factors, higher diabetes-related family conflict and lower adolescent diabetes self-management behaviors were associated with significantly higher caregiver DD.

**Conclusions:**

Correlates of caregiver DD provide insight into potential risks and modifiable factors that may help clinicians develop interventions to target caregiver DD to improve outcomes in both caregivers and adolescents with T1D.

Among youth under 20 years old, approximately 1 in 465 are diagnosed with type 1 diabetes (T1D) and onset is most common during adolescent years (Lawrence et al., 2021). Management of T1D requires an intensive and complex regimen, which includes frequently checking blood glucose levels and administering insulin, tracking carbohydrate intake, and carefully monitoring physical activity, yet most adolescents do not achieve the recommended glycemic target of hemoglobin A1c (HbA1c) < 7% (ElSayed et al., 2025; Foster et al., 2019). Adolescents with T1D not only face diabetes-related complications but are also at an increased risk of experiencing higher rates of depressive symptoms and anxiety compared to their peers without diabetes (Buchberger et al., 2016).

Along with the psychosocial and glycemic challenges associated with T1D, many adolescents experience negative emotions related to the burden of T1D management, known as diabetes distress (DD) (Fisher et al., 2019). Previous research has demonstrated a link between DD and higher HbA1c (a measure indicative of worse glycemic outcomes), lower health-related quality of life, and increased anxiety and depressive symptoms in adolescents (Hagger et al., 2016, 2018; Wasserman et al., 2021). Risk factors for DD in adolescents include higher HbA1c, less social support, and greater duration of T1D diagnosis (Hagger et al., 2016). In addition, DD may be more prevalent among female adolescents than male adolescents (Iturralde et al., 2019).

Caregivers of adolescents with T1D play a vital role in their child’s diabetes management. Their responsibilities include monitoring their child’s carbohydrate intake and exercise, tracking blood glucose levels, managing insulin intake via injections or pumps, and staying vigilant to promptly address episodes of hypoglycemia (ElSayed et al., 2025). Due to the role of the family in diabetes management, many caregivers also experience DD, which has been linked to greater symptoms of depression and reduced parent life satisfaction (Helgeson et al., 2012; Rumburg et al., 2017). Known risk factors for caregiver DD include family income, marital status, caregiver sex, and child age (Alkhatib et al., 2024; Hansen et al., 2012; Streisand et al., 2008).

Existing evidence also suggests that caregiver DD may have implications for adolescent psychosocial and glycemic outcomes, but the full extent to which caregiver DD might affect family functioning is still unclear. Guided by the Transactional Stress and Coping Model, which theorizes that the way caregivers cope with diabetes influences both parental and adolescent adjustment to the disease, we posit that caregiver DD may influence adolescent outcomes (Thompson & Gustafson, 1996). Previous studies have found a longitudinal relationship between parent DD and higher HbA1c levels in children ages 8–15, suggesting that caregiver distress may influence adolescent glycemic outcomes (Eilander et al., 2017). Caregiver DD has also been linked to reduced quality of life and depression in teens, both of which impact diabetes management (Abadula et al., 2024; Harrington et al., 2021; Hilliard et al., 2013). Finally, caregiver DD has been found to be associated with higher diabetes-related family conflict (Hessler et al., 2016), which is also linked to greater symptoms of depression and higher HbA1c in teens (Williams et al., 2009). Given the relationships between caregiver DD and adolescent outcomes highlighted above, it is important to understand DD in caregivers to promote optimal outcomes for both caregivers and their adolescent children with T1D.

The current study examined factors related to DD among caregivers of adolescents with T1D to (1) evaluate the prevalence of DD among caregivers of adolescents experiencing elevated DD, (2) examine demographic, family, and diabetes factors associated with caregivers’ DD, and (3) investigate which factors best explain the variance in caregivers’ DD. Based on prior studies highlighting risk factors for DD among adolescents, we assessed whether the same demographic factors (caregiver race/ethnicity, caregiver sex, adolescent age, time since T1D diagnosis, household income) and additional caregiver demographics (caregiver education, subjective social status) were associated with caregiver DD (Luo et al., 2021; Noser et al., 2019; Streisand et al., 2008; Vesco et al., 2018). We also examined associations between caregiver DD and family factors (diabetes-related family conflict and caregiver marital status) and diabetes-related factors (HbA1c, duration of T1D, and adolescent diabetes self-management). There is a need to identify both modifiable and non-modifiable factors to inform future screening and prevention efforts.

## Methods

### Participants

Adolescent and caregiver dyads were enrolled in a multisite randomized controlled trial aimed at evaluating the efficacy of a positive psychology text message-based intervention for adolescents with DD (clinical.gov identifier: NCT03845465) (Jaser et al., 2020). Adolescents were eligible for the study if they were ages 13–17 and diagnosed with T1D for ≥ 12 months. Adolescents completed the PAID-T, a validated measure used to assess DD in adolescents, to determine eligibility (Shapiro et al., 2018); adolescents were eligible if they reported at least moderate DD (PAID-T ≥ 34). Caregivers were eligible if they lived with the child at least 50% of the time, and caregiver DD was not a criterion for study inclusion. All participants gave informed consent, in line with the protocol approved by the Institutional Review Board (sIRB#191245). The current study is a secondary analysis of baseline data (*n* = 198 caregiver–adolescent dyads).

### Measures

Caregivers reported on demographics, including caregiver and adolescent racial and ethnic identity, caregiver and adolescent age, caregiver and adolescent sex, caregiver education level, caregiver marital status, and total household income. In addition, subjective social status (a measurement of an individual’s perceived position within the social hierarchy) was measured using the MacArthur Scale of Subjective Social Status (Adler et al., 2000). Race categorizations included White, African American/Black, Asian, American Indian or Alaska Native, Biracial, or Other. Ethnicity categorizations included Hispanic and non-Hispanic.

The Problem Areas in Diabetes-Parent of Teen version (P-PAID-T) is a 15-item validated measure used to assess DD among caregivers of adolescents with T1D (Shapiro et al., 2018). Caregivers rated the burden related to caring for an adolescent with T1D experienced within the past 6 months using a 6-point Likert scale (1 = *Not a problem*; 6 = *Serious problem*). Examples of items include, “Feeling ‘burned-out’ by the constant effort to manage my child’s diabetes,” “Feeling that my child does not check blood sugars often enough,” and “Feeling that I am often failing with managing my child's diabetes regimen.” P-PAID-T scores range from 15 to 90; clinically elevated scores ≥ 54. Cronbach’s α was 0.94 in the current sample.

The Revised Diabetes Family Conflict Scale (RDFCS) is a 19-item measure used to assess diabetes-related family conflict (Hood et al., 2007). Caregivers rated how often they argued with their adolescent over diabetes-related topics within the past month using a 3-point Likert scale (1 = *Never argue*; 2 = *Sometimes argue*; 3 = *Always argue*). Examples of items include, “During the past month I have argued with my child about remembering to check blood sugars,” and “During the past month I have argued with my child about carrying sugar/carbs for reactions.” Potential RDFCS scores range from 19 to 57; higher scores reflect higher levels of diabetes-related family conflict. Cronbach’s α = 0.92 in the current sample.

The updated Self-Care Inventory is a measure assessing diabetes self-management behaviors that reflects contemporary diabetes management (Jaser et al., 2025). In the current study, caregivers rated how often their adolescents engaged in diabetes self-management behaviors on a 5-point Likert scale (1 = *Never do it*; 5 = *Always do this as recommended without fail*). A mean score was used for analysis; higher scores reflect greater adolescent self-care. Cronbach’s α = 0.74 in the current sample.

Trained members of the research team from each study site extracted HbA1c data from the adolescents’ electronic medical records (point-of-care values) and at-home HbA1c kits (Coremedica^TM^) (for adolescents who had telehealth diabetes visits). The child’s most recent HbA1c value was used for data analysis, which was typically collected the same day as study surveys were completed. One hundred eighty-eight participants had HbA1c values that were used for analysis (*M *= 9.1; *SD *= 2.1); several participants did not have an HbA1c value available due to COVID-19-related disruptions to clinical care.

### Data analysis plan

The current study is a secondary analysis of baseline data (data will be shared upon reasonable request to the corresponding author). Using IBM SPSS v28, analyses were conducted to assess the correlates of caregiver and adolescent demographic factors, family characteristics, and diabetes factors with caregiver DD. Caregiver DD has skew and kurtosis within the acceptable range, and therefore, parametric tests were used. Bivariate analyses, independent samples *t*-tests, and analysis of variance were used to evaluate the relationship between demographic factors and caregiver DD. Finally, stepwise linear regression analysis was used to examine which demographic variables best explained the variance in caregiver DD. Variables included in the analysis were derived from previous studies of DD (income, marital status, adolescent age, caregiver sex), and a correlation >0.2, indicating at least a small effect (Cohen, 1988). With a sample size of 198, we had a power of .99 to detect a medium effect size in the regression analysis. A *p* value < .05 was used to determine the significance of our analyses.

## Results

Caregiver demographic characteristics are listed in Table 1. Caregivers had a mean DD score of 49.3 (*SD *= 17.6), with 39.1% (*n *= 78) of caregivers of adolescents with T1D reporting clinically elevated DD symptoms (score of ≥54).

**Table 1.** Caregiver characteristics.

Demographic Characteristics	Caregiver age, years (*mean* [*SD*])	45.7 [7.3]
	**Caregiver sex, female (*N* [%])**	169 [86%]
	**Caregiver relationship, *N* [%]**	
	Biological parent	186 [94%]
	Stepparent	1 [<1%]
	Grandparent	5 [3%]
	Other family member	4 [2%]
	Other	2 [1%]
	**Caregiver race, *N* [%]**	
	White	137 [70%]
	African American/Black	41 [21%]
	Asian	9 [5%]
	American Indian or Alaska Native	2 [1%]
	Biracial	1 [<1%]
	Native Hawaiian or Pacific Islander	5 [3%]
	Other	3 [2%]
	**Caregiver ethnicity, *N* [%]**	
	Non-Hispanic	188 [93%]
	Hispanic	7 [4%]
	Not indicated	3 [2%]
	**Caregiver education, *N* [%]**	
	Less than high school	3 [2%]
	GED/high school graduate	26 [13%]
	Trade/technical school or some college	48 [24%]
	College graduate	68 [34%]
	Graduate school	52 [26%]
	Not indicated	1 [<1%]
	Subjective social status (*mean* [*SD*])	63.8 [18.1]
	**Household income**, ***N* [%]**	
	<$24,999	16 [8%]
	$25,000–$49,999	35 [18%]
	$50,000–$89,999	47 [24%]
	$90,000–$149,999	42 [21%]
	≥$150,000	56 [28%]
	Not indicated	2 [1%]
	**Marital status, *N* [%]**	
	Married/partnered	150 [76%]
	Single	19 [10%]
	Divorced/separated	25 [13]
	Widowed	4 [2%]

*Note.* Percentages rounded to the next whole number. In some cases, the total does not equal 100%.

### Demographic factors and caregiver DD

Independent samples *t*-tests revealed significant differences in caregiver DD related to sex; female caregivers reported significantly higher DD (*M *= 50.3; *SD = *17.6) than male caregivers (*M *= 42.2; *SD *= 16.0), *t*(194) = −2.28, *p* = .02. Income was dichotomized (1 = household income <$90,000 per year; 2 = household income ≥$90,000 per year) in the model based on the median household income (U.S. Census Bureau, 2022), and 49% of our sample had a median household income of $90,000 or more. An independent samples *t*-test indicated that caregivers with lower household income reported significantly more DD (*M *= 52.8; *SD *= 18.1) than caregivers with higher household income (*M *= 45.9; *SD* =16.5), *t*(193) = 2.80, *p* = .006. As seen in Table 2, bivariate correlations indicated that lower subjective social status was significantly associated with higher levels of caregiver DD (*r *= −.24) and that DD was higher among caregivers of younger adolescents (*r *= −.20). No significant differences were observed between caregiver DD and caregiver race or ethnicity, age, or educational level.

**Table 2.** Correlations between caregivers’ diabetes distress, demographic, family and diabetes-related characteristics.

	1.	2.	3.	4.	5.	6.	7.	8.
P-PAID-T	—							
Caregiver age	−.11	—						
Adolescent age	−.20**	.22**	—					
Subjective social status	−.24***	.41***	−.12	—				
RDFC	.54***	−.05	.00	−.25***	—			
Diabetes duration	.07	.03	.18*	−.17*	.00	—		
SCI-U	−.39***	−.07	−.01	.12	−.18*	−.20**	—	
HbA1c	.23***	−.07	−.04	−.41***	.25***	.17*	−.30***	—

*Note*. *P-PAID-T* = Parent Problem Areas in Diabetes Teen; *RDFC* = Revised Diabetes Family Conflict; *SCI-U* = Self-Care Inventory Updated.

* *p *< .05.

** *p* < .01.

*** *p* < .001.

### Family characteristics and caregiver DD

Marital status was dichotomized into single/widowed/divorced or married/partnered, and partnered caregivers reported significantly lower DD (*M *= 47.9, *SD *= 16.8) than nonpartnered caregivers (*M *= 53.6, *SD *= 19.3) (*p* = .05). Finally, bivariate correlations revealed that higher caregiver reports of diabetes-related family conflict were significantly associated with higher caregiver DD (*r* = .54).

### Diabetes factors and caregiver DD

As seen in Table 2, bivariate correlations indicated that higher levels of caregiver DD were associated with significantly higher HbA1c values (*r* = .23) and lower diabetes self-management in adolescents (*r *= −.39). Length of time since T1D diagnosis was not significantly associated with caregiver DD.

### Linear regression analyses

In the first step of the model, we included demographic characteristics (caregiver sex, adolescent age, household income, and caregiver-reported subjective social status). In the next step of the model, we added family characteristics (caregiver marital status and caregiver-reported diabetes-related family conflict). In the final step of the model, we added diabetes factors (HbA1c and caregiver-reported adolescent diabetes self-management). The regression model was significant, explaining 50% of the variance in caregiver DD (*R^2^* = .50; *F*(8,151) = 18.7; *p* < .001) (Table 3). In the final model, adolescent age (*β* = −0.22), caregiver-reported diabetes-related family conflict (*β  *= 0.50), and caregiver-reported adolescent diabetes self-management (*β* = −0.27) were significantly associated with caregiver DD (all *p* < .001).

**Table 3.** Linear regression analyses.

Variable	*R* ^2^	*B*	*β*	*p*	95% CI
	.50				
Caregiver sex (1)		3.40	.07	.257	[−2.51 to 9.31]
Adolescent age		−2.86	−.22	<.001	[−4.39 to −1.33]
Household income (1)		−1.51	−.04	.568	[−6.71 to 3.70]
Caregiver-reported subjective social status		−0.09	−.08	.261	[−0.25 to 0.07]
Caregiver marital status (1)		2.54	.06	.403	[−3.44 to 8.51]
Caregiver-reported diabetes-related family conflict		1.17	.50	<.001	[0.88 to 1.46]
Caregiver-reported adolescent diabetes self-management		−7.91	−.27	<.001	[−11.48 to −4.35]
Adolescent HbA1c		0.14	.02	.818	[−1.02 to 1.29]

*Note.* Dependent variable: caregiver DD. Caregiver sex: 1 = female, 2 = male. Caregiver marital status: 1 = single, widowed, or divorced; 2 = married or partnered. Household income: 1 <$90,000 per year; 2 ≥$90,000 per year.

## Discussion

The current study examined demographic, family, and diabetes factors associated with DD among caregivers of adolescents with T1D who were experiencing elevated DD. This examination is one of the first to assess factors related to experiences of DD among caregivers of adolescents with T1D. To support the clinical application of these findings, analyses should be interpreted as potential risk factors and modifiable factors to support (1) the identification of caregivers who may be at increased risk of experiencing DD and (2) the identification of modifiable factors that may be altered through evidence-based behavioral interventions and reduce caregiver DD.

Nearly 40% of the caregivers in our sample reported experiencing clinically elevated DD. This is a somewhat higher occurrence of caregiver DD than identified in other studies of caregivers of youth with T1D; for example, Abadula and colleagues (2024) found significant DD in 35% of caregivers, and Alkhatib and colleagues (2024) found significant DD in 27% of caregivers. Our higher rates may be due to the eligibility criteria for the study, which enrolled adolescents who were experiencing elevated DD. Additionally, caregiver DD was higher among female caregivers than male caregivers, in line with previous literature finding a higher prevalence of distress among mothers compared to fathers with T1D (Whittemore et al., 2012), and a higher prevalence of DD among female adolescents with T1D (Inverso et al., 2022). These findings may be best explained by the additional burdens of managing diabetes that are placed on mothers/female caregivers who assume a large portion of diabetes management responsibilities. Further, caregiver DD was associated with child age, such that caregivers of younger adolescents reported higher DD, similar to findings in prior studies (e.g., Alkhatib et al., 2024). This difference may be due to larger burdens of diabetes management experienced by caregivers, as younger adolescents may have less responsibility for their diabetes management due to their developmental stage. Additionally, lower household income and subjective social status were associated with higher caregiver DD. Disparities in caregiver DD based on income further highlight the burden experienced by caregivers due to the cost of diabetes supplies and may contribute to health inequities (Pfiester et al., 2021). These findings have clinical relevance as they pinpoint potential risk factors for caregiver DD.

In terms of family characteristics, analyses revealed a significant difference in caregiver DD related to marital status, such that single/unpartnered caregivers reported higher DD. In line with previous literature (e.g., Eshtehardi et al., 2021), this result highlights the potential impact of caring for an adolescent with T1D without the support of a partner. Although we cannot draw conclusions about the relational status of unmarried caregivers based on the available data, unpartnered caregivers may oversee the majority of diabetes-related responsibilities without social support from other family members. Previous literature has demonstrated that caregivers with social support have better psychosocial functioning (Thorsteinsson et al., 2017). Consistent with previous studies (Williams et al., 2009), higher diabetes-related family conflict was also associated with higher caregiver DD. Diabetes-related family conflict and caregiver DD are likely bidirectional, as others have posited (Helgeson et al., 2012; Williams et al., 2009). It is possible that the burdens of caring for an adolescent with T1D may lead to caregiver DD, and distressed caregivers may employ maladaptive strategies (e.g., inefficient communication, punitive reinforcement) that influence diabetes-related family conflict (Jaser & Grey, 2010). Alternatively, it is plausible that diabetes-related family conflict around problems with diabetes management may precipitate caregiver DD. Though causal relationships cannot be drawn from the current cross-sectional study, the relationship between these constructs is likely multifactorial and unique to each family unit.

In terms of diabetes-related factors, after adjusting for demographic factors, adolescents’ self-management behaviors were negatively associated with caregiver DD, but the association with HbA1c was no longer significant. This is in contrast with previous literature demonstrating a link between caregiver DD and youth HbA1c (Alkhatib et al., 2024; Shapiro et al., 2018). It may be that caregivers experience greater distress when adolescents are not engaging in self-management behaviors. Further investigation into these relationships may strengthen the clinical relevance and support the development of family-based interventions to improve adolescent diabetes management and caregiver DD.

### Strengths

The current study has many strengths, including a relatively large sample from two pediatric diabetes clinics. Historically, research has focused on parental well-being, specifically that of biological mothers, and does not represent all family archetypes. The current sample extended inclusion to additional types of caregivers (e.g., fathers, stepparents, grandparents, legal guardians, other family members, etc.) and brings a representative approach to caregiver well-being, especially in different types of households. Additionally, our sample included a higher percentage of Black non-Hispanic participants compared to existing studies of children with T1D, making our findings particularly relevant to this group (Jaser et al., 2008; Pettitt et al., 2014).

### Limitations

Despite the strengths identified above, the current study lacks representation of Hispanic participants and caregivers without secondary education compared to other studies of adolescents with T1D (Pettitt et al., 2014). Additionally, while we assessed the caregiver DD, data were not available on the caregiver’s mental health status, which may exacerbate DD symptoms. We also did not assess system-level factors that may contribute to caregiver DD, such as inaccessibility of diabetes supplies due to problems with health insurance, which are likely to impact caregiver DD and further contribute to disparities within diabetes care (Kahkoska et al., 2021).

### Future directions

Research on caregiver DD is limited and continued exploration of contributors to caregiver DD is warranted. The current study solely focused on correlates of caregiver DD cross-sectionally, which prevents causal inferences, Therefore, future research should explore the associations between adolescent and caregiver DD with, glycemic outcomes, adolescents’ self-management behavior, and diabetes-related family conflict with longitudinal studies to better understand how DD is experienced in a family/household dynamic over time. These findings may also guide the development and implementation of dyadic interventions to decrease diabetes-related family conflict and alleviate experiences of caregiver DD, such as pilot intervention studies conducted by Jaser and colleagues (2018) and Alkhatib and colleagues (2025).

This study further highlights the importance of diabetes healthcare providers identifying families experiencing the negative effects of DD, and the implementation of standardized DD screening in diabetes clinics, including caregivers as well as youth. Awareness of DD by healthcare professionals may connect more families with social programs that can minimize the financial impact of managing T1D to reduce caregiver DD. For example, joining single parents and those from lower-income households with a peer mentor may alleviate some of the burdens of diabetes management (Tully et al., 2017), and interventions to address caregiver DD show promise (Alkhatib et al., 2025; Jaser et al, 2018). Future investigation into other systemic factors that impact diabetes management may help clinicians and researchers better understand the influences of caregiver DD.
